# Single but Not Combined In Vitro Exposure to Bisphenol A and Nanoplastics Affects the Cholinergic Function of the Ascidian *Ciona robusta*

**DOI:** 10.3390/jox14040103

**Published:** 2024-12-05

**Authors:** Safa Melki, Emma Ferrari, Raja Ben Ahmed, Antonietta Spagnuolo, Ilaria Corsi

**Affiliations:** 1 Department of Biology, Laboratory of Ecology, Biology and Physiology of Aquatic Organisms LR18ES41, Faculty of Sciences of Tunis, University of Tunis El Manar, Tunis 2092, Tunisia; safa.melki@etudiant-fst.utm.tn (S.M.); raja.benahmed@fst.utm.tn (R.B.A.); 2 Department of Physical, Earth and Environmental Sciences, University of Siena, 53100 Siena, Italy; ilaria.corsi@unisi.it; 3 Department of Biology and Evolution of Marine Organisms, Stazione Zoologica Anton Dohrn, 80121 Naples, Italy; niettaspagnuolo1@gmail.com

**Keywords:** ecotoxicology, cholinesterases, ascidians, emerging contaminants, mixture toxicity, polystyrene nanoparticles

## Abstract

Nanoplastics are known to represent a threat to marine ecosystems. Their combination with other contaminants of emerging concerns (CECs) may amplify ecotoxic effects, with unknown impacts on marine biodiversity. This study investigates the effects, single and combined, of bisphenol A (BPA)—one of the most hazardous CECs—and polystyrene nanoparticles (PS NPs)—as a proxy for nanoplastics, being among the most commonly found asmarine debris—on cholinesterase (ChE) activities of the ascidian *Ciona robusta*. ChE activity was first measured in the siphons, tunic, and viscera of wild-caught adult specimens and exposed in vitro to BPA (0.01, 0.21, 0.69 mM) and PS NPs (0.0096–0.096 mM; 8.096 × 10^9^–10^10^ particles, respectively) alone and combined for 15 min of incubation. PS NPs’ behavior in milliQ water and in the ChE assay reaction buffer was characterized alone, combined with BPA, and analyzed through ζ-potential measurements via Dynamic Light Scattering. The results revealed that ChE activity was predominant in the viscera and siphons of *C. robusta*; PS NPs did not affect the ChE activity alone or combined, while BPA caused a concentration-dependent inhibition of ChE activity in the viscera. No changes in ζ-potential were observed for PS NPs alone or combined with BPA in the ChE buffer, suggesting no interaction. Further investigations are needed to understand the potential neurotoxic consequences for *C. robusta* and ecological risk scenarios due to exposure to BPA and nanoplastics in marine coastal waters.

## 1. Introduction

Cholinesterases (ChE), and in particular acetylcholinesterase activity (AChE), are widely used as biomarkers of neurotoxicity in marine pollution monitoring and assessment due to their conservative role in hydrolyzing the neurotransmitter acetylcholine in the peripheral and central nervous systems of many organisms [1]. Studies have demonstrated that exposure to certain compounds, including industrial chemicals and plastic additives, causes AChE inhibition in marine species, highlighting the sensitivity of this biomarker also for monitoring the effects of contaminants of emerging concern (CECs) [2,3]. A wide range of toxic chemicals can potentially inhibit AChE activity across diverse species, from invertebrates to mammals, thus impairing neurotransmission and embryo development [4,5]. Bisphenol A (BPA) is among the most hazardous CECs associated with domestic sewage effluents and it is widely used in the production of epoxy resins and polycarbonate plastics [6]. BPA is nearly ubiquitous in aquatic environments, with a strong association with plastic waste [7]. Data on the environmental fate of BPA—as well as its levels in environmental matrices—are scarce as compared to data on its biological effects, which include a broad range of biological functions, such as phenotypic abnormalities, altered behavior and disruption in the cardiovascular and reproductive system, with consequences on development, growth, and survival of marine species [8,9,10]. The biological effects of BPA are most likely mediated by its endocrine-disrupting action—particularly as an estrogen-mimic—thus impacting development, metabolism, and the reproductive system, but also affecting the central nervous system (CNS) [11]. Recent findings on BPA as a neurotoxicant revealed that the bioaccumulation in the zebrafish brain causes overexpression of myelin (MBP), with significant inhibition of AChE activity [3]. Therefore, AChE is a target of BPA also in *Ciona* and merits further investigation also in realistic exposure scenarios when mixtures of CECs are present and affect biological responses as well as pathways of ecotoxicity. Nanoplastics are listed among CECs and being abundant in the marine coastal waters of the Mediterranean Sea, they can interact with other micropollutants released into wastewater treatment plants’ effluents, including plastic additives such as BPA. As such, nanoplastics and BPA can interact, leading to synergistic, antagonistic, or additive effects and thus affecting several biological functions of marine species [12,13,14]. Current evidence suggests that plastics, regardless of whether they are micro or nano size, play a substantial role in exposing marine organisms to complex mixtures of chemical contaminants [15,16,17]. The neurotoxicity of nanoplastics and BPA single and/or combined has been overlooked in marine species, although exposure to them occurs in marine coastal areas. We recently demonstrated the absence of interaction between BPA and nanoplastics (polystyrene nanoparticles, PSNPs) in natural seawater, using as an end point the development of *Ciona robusta* embryos. While BPA alone caused a reduced pigmentation of sensory organs (4.5 µM and 10 µM), in agreement with previous findings [18,19,20], no effect was observed by PS NPs at the range of tested concentrations (1 and 10 g/mL) either alone or combined with BPA [21]. The ability of salts and natural organic matter (NOM) present in natural seawater (NSW) were considered responsible for limiting BPA adsorption onto PS NPs, as also demonstrated by the significant changes observed in the ζ-potential, which was far less negative upon incubation with NSW. Indeed, the ζ-potential is a measure of the electrical potential at the boundary between the particle and the water suspension; therefore, it represents a crucial indicator of the stability of colloidal dispersions, here applied to PS NPs in NSW.

The ascidian *Ciona* spp.—at the embryo, juvenile, and adult stages—is increasingly used in ecotoxicological studies, thanks to its phylogenetic position as a sister group of vertebrates [22]. Adults have been used to monitor anthropogenic stressors such as heavy metals, MPs, phthalate acid esters, and pharmaceutically active compounds [23,24] while embryos and juveniles have been instrumental in addressing nanoplastic ecotoxicity [19,25]. A remarkable increase in AChE activity has been detected after the exposure of unfertilized eggs, embryos, and juveniles of *Ciona* to tributyltin—a toxic organotin compound used primarily as an antifouling agent—suggesting that this enzyme is targeted by it [26,27].

Our study aims to characterize ChEs’ activity across different tissues of *Ciona robusta* and assess the in vitro effects of BPA and PS NPs, single and combined. An in vitro study was performed using a range of concentrations (0.01–0.21–0.69 mM for BPA and 0.0096–0.096 mM for PS NPs) resembling realistic and acute exposure scenarios and to align with previous investigations on embryotoxicity [21].

## 2. Materials and Methods

### 2.1. Materials

Unfunctionalized 20 nm PS NPs were purchased from Bangs Laboratories Inc. (Fishers, IN, USA) and received as stock suspensions (100 mg/L) in deionized water. To resemble realistic exposure scenarios to PS NPs in the marine environment, an unfunctionalized batch was tested here. A brief 15 min bath sonication at 70% power was performed on the stock suspensions to ensure optimal dispersion and prevent aggregation. Subsequently, working suspensions of PS NPs were prepared at concentrations of 1 mg/L and 10 mg/L in MilliQ water (mQW) and stored in sterile vials at 4 °C until use. PS NPs’ stock characterization in mQW was performed by Dynamic Light Scattering (DLS) and already reported by Ferrari et al. [21] (ζ-Average (nm) 22.8 ± 0.3 nm, PDI 0.35 ± 0.4, ζ-Potential (mV) 51 ± 5 mV). Here, the ζ-potential (mV) of PS NPs aliquot alone and combined with BPA in mQW and in the ChE reaction buffer (0.1 M Na_2_PO_4_, pH 7.2, Ellman et al. [28]) used for in vitro ChE analysis was measured to infer potential interaction between particles and BPA in the exposure media. The measurements were carried out using 50 µg/mL of PS NP and 0.69 mM of BPA using a Zetasizer Nano ZS90 (Malvern, Malvern, UK) equipped with the Zetasizer Nano Series software (Version 7.02). BPA was purchased from Merck (CAS Nr. 80-05-7) and dissolved in mQW after a bath sonication (10 min, 70% power) at 50 mg/L, 100 mg/L, and 300 mg/L stock solutions. A standardization of units for BPA and PS NPs was conducted using molarity based on their respective molecular weights (BPA: 228.29 g/mol, Styrene: 104.15 g/mol) and the number of NPs/mL has been calculated for both concentrations of PS NPs (see Appendix A—Supplementary information). So, the BPA range of tested concentration was: 0.01–0.21–0.69 mM; PS NP 0.0096–0.096 mM and 8.096 × 10^9^–8.096 × 10^10^ particles. Acetylthiocholine (iodide) (ASCh), 5,5′-Dithio-bis-(2-nitrobenzoic Acid) (DTNB), and selective inhibitor of AChE 1,5-Bis(4-allyldimethylammoniumphenyl) pentan-3-one dibromide (BW284c51) (Sigma, St. Louis, MO, USA).

### 2.2. Ciona robusta

Adult specimens of ascidian *Ciona robusta* were collected in the Gulf of Taranto (Italy) by local fishermen between March and June 2023 and immediately shipped in cool boxes to the University of Siena (Italy). Animals were kept in the aquarium for 1 week in NSW collected from the Tuscany coast of the Mediterranean Sea (18 ± 1 °C, salinity 40 ± 1‰, dissolved O_2_ 7 mg/L, pH 8.1), specimens were kept under constant aeration and photoperiod (16:8, light/dark).

### 2.3. AChE Activity Characterization

Three types of tissues as inner body organs (branchial sac, heart, viscera and gonads (V), tunic (T), and siphons (S)) of 51 adult specimens were carefully dissected and stored separately in 1.5 mL Eppendorf for ChE activities characterization. Tissues (V, S, T) were finely cut with scissors while kept on ice and homogenization buffer (Low Salt Triton, 20 mM Tris, 5 mM MgCl_2_, 0.1 mg/mL Bacitracin, 8 × 10^−3^ TIU/mL Aprotinin, 1% Triton X-100, pH = 7.4) was added in weight/volume (*w*/*v*) ratios as 1:5: 0.1 g: 0.5 mL. Samples were homogenized using a Potter–Elvehjem and centrifuged at 8400× *g* and 4 °C for 20 min. The pellet was then discarded, and the supernatant obtained from V, S, and T was used for the measurement of ChE activities; preferably fresh or upon storage at −80 °C. The AChE activity was assayed by the method of Ellman et al. [28], modified for a microplate reader.

ASCh was selected as a preferential substrate to determine the optimum concentration for the measurement of true-AChE activity. Initial assay conditions in the reaction mixture (final volume 300 µL) were as follows: 0.1 M ChE reaction buffer (0.1 M Na_2_PO_4_ pH 7.2), supernatant (V, S, and T), DTNB (1 mM), and ASCh substrate (1 mM). Each sample was evaluated in three replicates. The reaction starts when the substrate is added and read for 5 min. The amount of supernatant and ASCh was changed to obtain a better linearity of the reaction curve as follows: four different amounts of supernatant (40, 60, 80, and 100 µL) and three amounts of ASCh (10, 15, and 20 µL) were tested. Reaction rates were measured using a BIO-RAD max tunable microplate reader (Model550) (BioRad Laboratories, 2000 Alfred Nobel Drive, Hercules, CA) which measured the rate of change of absorbance at 405 nm for 5 min after the addition of substrate at 20 °C. ChE vs. ASCh activities were initially expressed as Δ absorbance units/min, converted to nmoles hydrolyzed substrate/min and normalized by tissue total protein content (mg).

### 2.4. Acetylcholinesterases Activity In Vitro Study

The inhibition studies were carried out by incubating for 15 min the reaction mixture (as a modification of the Ellmann method [28]) (supernatant V, S, and T; ASCh, DTNB) with BPA and PS NPs single and in combination at the following concentrations: BPA 0.01 mM, 0.21 mM, and 0.69 mM, PS NPs 1–10 mg/L (respectively, 0.0096 mM and 0.096 mM and 8.096 × 10^9^–8.096 × 10^10^ particles), PS NPs (0.096 mM) combined with BPA (0.69 mM). BW24C51 was used to confirm true-AChE activity as a selective inhibitor of pure AChE by adding 20 µL (1 μM final reaction concentration) to the reaction mixture and incubating for 15 min.

To determine the inhibition of BPA, the stock solutions were diluted in the reaction buffer to reach the respective concentrations (0.01–0.21–0.69 mM) and added to the mixture as follows: 160 µL 0.1 M Reaction buffer (pH 7.2) and BPA, supernatant 100 µL, 20 µL DTNB (1 mM), and 20 µL of ASCh substrate (1 mM); incubating for 15 min. The same procedure was carried out for PS NPs (0.0096–0.096 mM). To carry out the combined exposure in vitro, PS NPs (0.096 mM) and BPA (0.69 mM) were diluted in the same vial with reaction buffer and then added to the mixture, following the same process as described earlier. Each sample was analyzed in three replicates and, subsequently, the mean was calculated among samples subjected to the same treatment to obtain a representative value for each treatment. The total protein concentration of extracts of *Ciona* tissues (T, S, and V) was measured following the method of Bradford [29] using bovine serum albumin as standard; values are expressed in mg total protein per ml supernatant.

### 2.5. Statistical Analysis

Data are shown as mean ± standard deviation. Statistical analysis was performed using GraphPad Prism software (Version 8.0.1, San Diego, CA, USA) using 2-way ANOVA with a Tukey multiple comparisons test with significance indicated in the figures as * *p* < 0.0001 and Unpaired *t*-test, significance as * *p* < 0.01. A Pearson correlation test was used to analyze concentration-dependent variables in the Ellman assay.

## 3. Results

### 3.1. Behavior of PS NPs in mQW and Combined with BPA in ChE Reaction Buffer

DLS analysis on the ζ-potential of PS NPs was performed on NPs alone in mQW and combined with BPA in ChE reaction buffer solution at time 0 and after 15 min, to resemble the in vitro incubation media and time (Table 1). PS NPs showed a negative surface charge in mQW (−51 ± 5 mV) (Table 1) and similar values were also measured in those suspended in ChE reaction buffer either alone (−49.1 ± 1.78 mV) and with BPA at T_0_ (−50.8 ± 1.72 mV) and after 15 min of incubation T_15_ (−47.3 ± 1.57 mV).

### 3.2. Characterization of ChE Activity in Ciona Tissues

**Figure 1:**
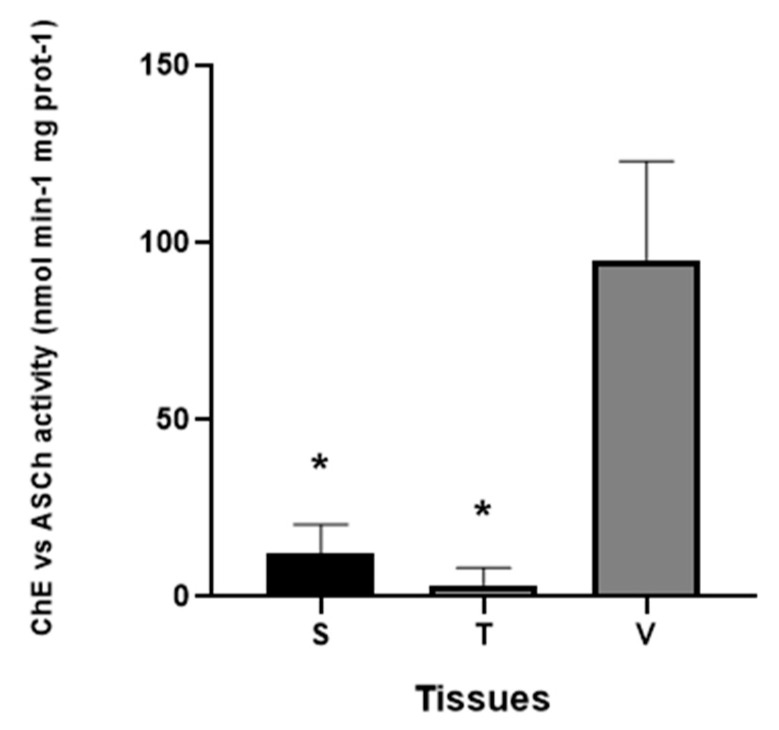
ChE vs. ASCh activity (nmol/min/mg prot) in the three tissues of Ciona as siphons (S), tunic (T), and viscera (V). Values are shown as mean ± standard deviation. Asterisks denote significant differences compared to (V) group. A 2-way ANOVA with a Tukey multiple comparisons test was performed.

As a first step, ChE vs. ASCh activities on viscera (V), tunic (T), and siphon (S) were assessed. The findings indicate that the viscera (V) of *Ciona* exhibited the highest ChE activity vs. ASCh compared to tunic (T) and siphon (S) (Figure 1) (* *p*-value < 0.0001). Only in V and S, variations in substrate ASCh concentration (0.24 mM to 0.48 mM) and the amount of tissue extract (40 µL–100 µL) significantly affected ChE activities with a similar pattern (Table 2); however, activities of T extract did not vary (Table 3). Moreover, by increasing the amounts of the samples of V extracts (ranging from 40 to 100 µL) and substrate (0.24 and 0.48 mM), ChE vs. ASCh significantly increased. The Pearson correlation coefficient “r” was 0.94 and 0.99, respectively, indicating a clear and proportional correlation (Table 3). The use of the selective inhibitor BW284C51 reveals that ChE vs. ASCh activities in V were mostly of AChE, since the inhibition observed was 84.36% of the total activity measured without the inhibitor (Table 2).

### 3.3. In Vitro BPA and PS NP Exposure Study

**Figure 2:**
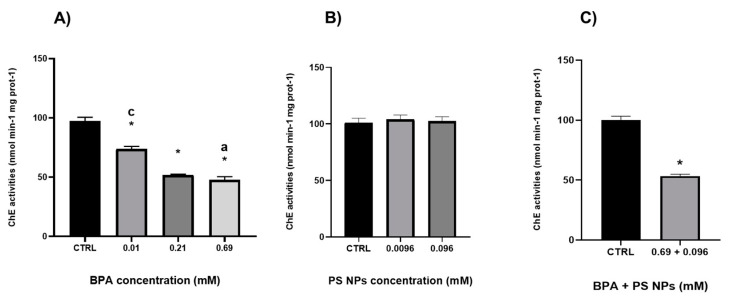
ChE vs. ASCh activity (**A**) = BPA alone, (**B**) = PS NPs alone, (**C**) = PS NPs + BPA combined) in the viscera of Ciona in vitro exposed to BPA (0.01–0.21–0.69 mM), PS NPs (0.0096–0.096 mM) and combined (0.096 mM PS NPs and 0.69 mM BPA). Values are shown as mean ± standard deviation. Asterisks denote significant differences compared to control groups and letters between treatment groups (a = 0.01 mM, c = 0.69 mM). Two-way ANOVA with a Tukey multiple comparisons test and anUnpaired *t*-test were performed.

Figure 2A shows the inhibition of ChE vs. ASCh activity caused by BPA in viscera (V) extracts of *Ciona*. A clear concentration-dependent inhibitory effect was observed, with a significant decrease in ChE vs. ASCh activity as the BPA concentration increased. This decrease is already observable at the lowest BPA concentration (−30%; 0.01 mM) compared to the control group. All tested concentrations significantly decreased ChE vs. ASCh activity in V extracts compared to the control (* *p* < 0.0001), with an inhibition percentage of approximately 50% for 0.21 mM and 0.69 mM BPA. Conversely, exposure to PS NPs at both molarities (0.0096 and 0.096 mM) did not cause any inhibition in ChE vs. ASCh activity (Figure 2B) with values such as unexposed tissue extracts. Combined exposure to PS NPs and BPA, both administered at their highest doses (0.096 mM and 0.69 mM, respectively), caused a significant inhibition (* *p* < 0.01) compared to control (Figure 2C). This inhibition was notably like the effect observed with BPA alone at the intermediate and the highest concentration tested (Figure 2A). Table 4 presents the percentage of inhibition for each concentration tested on ChE vs. ASCh activity.

## 4. Discussion

This study aims to characterize for the first time the ChE vs. ASCh activity in different tissues of *Ciona* and investigate the in vitro sensitivity towards BPA and PS NPs alone and in combination. ChE vs. ASCh activities were mainly identified in viscera and siphons, while the tunic exhibited bare activity close to the detection limit. The almost complete inhibition of ChE vs. ASCh activity upon incubation with the selective AChE inhibitor (BW284C51) revealed the presence of true-AChE activity in both viscera and siphons of adults of *Ciona*. Our findings are consistent with the knowledge that AChE in ascidians has a pivotal role in neuromuscular synapses but is also found on the external surfaces of cells and in various cytoplasmic regions [30]. At the embryonic stages, AChE is involved in labeling embryonic muscle cells [31] and larval adhesive papillae, playing a crucial role in the development of various tissues, while in adults its main role is in regulating muscle and ciliary activities [30,32]. The study by Arkett et al. [32] indicated that ChE activity in the adult and juvenile stages of ascidians is primarily situated in the innervation of the branchial basket and is not restricted to synaptic sites but is distributed throughout the neurons. Recently, AChE histochemical reactions, on fresh preparations of adult pharyngeal gills of *Ciona*, revealed strong signals on the laterodistal ciliated cells of stigmata, referred to as trapezial cells. The direct administration of ACh and other agonists of nicotinic ACh receptors (nAChRs) onto ciliated cells reliably evoked ciliary arrest that persisted for seconds in a dose-dependent manner. Furthermore, the authors provided evidence to show that a nicotinic ACh receptor, A7/8-1 nAChR, expressed in *Ciona* stigmata, mediates neuro-ciliary transmission to elicit ciliary arrest [33]. This is consistent with our findings, showing AChE activity in the viscera including the large branchial sac. Furthermore, Zaniolo et al. [34] confirmed the presence of numerous free nerve endings in the oral siphon tentacles, while describing the development of the nervous system in ascidians. Moreover, the study reported no innervation running through the tunic, supporting our findings, as no activity was detected in the tunic extracts. ChE vs. ASCh activities increased proportionally with the amount of siphon extracts while keeping constant substrate molarity (0.24 mM). In contrast, tunic extracts showed a certain variability, with a significant increase of ChE vs. ASCh activities from 40 to 80 µL (11.6 ± 2.2) but a decrease at 100 µL (0.18 ± 0.6). This variability makes uncertain the hydrolysis of ChE vs. ASCh in the tunic extracts played by the only AChE enzyme (Table 3). More recently, the entire neural network of *Ciona* has been visualized in adult transgenic animals harboring a reporter gene construct driven by the pan-neuronal PC2 promoter [35]. The data clearly showed that eight main anterior nerves and ten main posterior nerves, leading from the cerebral ganglion, head toward the oral siphon and atrial siphon, and periphery branch into thin nerves that reach the edge of the siphon lobes. Thus, the innervation surrounding the siphons is complex; however, as the authors point out, further studies will allow us to identify the neuropeptides and neurotransmitters that are likely to regulate the functions of these nerves. Our findings further support such hypotheses, as indicated by lower cholinergic activity in the siphons compared to the viscera, suggesting variations in neural activity based on anatomical regions and peptidergic regulatory networks. BPA was revealed to be a concentration-dependent inhibitor of ChE vs. ASCh activity in *Ciona* viscera and in a concentration-dependent manner. At environmentally realistic concentrations, BPA has already been reported to affect the embryo development of aquatic species, including invertebrates [7,19,36]. Regarding ascidians, BPA is probably the individual chemical that has been most addressed in emerging contaminants’ ecotoxicological studies. Messinetti et al. [18] reported that larvae of *Ciona* and *Phallusia mammillata* exposed to BPA, showed short and kinked tails, impaired neural development and malformations of pigmented organs (otolith and ocellus). Moreover, Gomes et al. [19] reported that micromolar doses of BPA inhibited otolith movement within the sensory vesicle and that BPA may target ERRs (Estrogen-Related Receptors) during otolith movement in *P. mammillata*. Together, these observations suggest that BPA may affect ascidian otolith differentiation by altering ERR activity, whereas otolith pigmentation defects might be due to the known inhibitory effect of bisphenols on tyrosinase enzymatic activity. Similarly, BPA can cause decreased pigmentation in zebrafish embryos (5.0 mgL^−1^) by downregulating the melanin synthases and reducing the melanin content. The role of BPA as a ligand of the zebrafish tyrosinase (Tyr) family of proteins has been hypothesized, thus causing skin pigmentation interference [20]. These studies align with our previous findings, confirm that one of the most reproducible phenotypes of BPA in ascidian larvae is the disruption of the pigmented cells [21]. The results presented here reveal that BPA can affect not only the most vulnerable stages of *Ciona*, such as embryo or larvae, but also the neurotransmission system of the adult stage, impairing cholinergic activity. These findings are consistent with those obtained recently on zebrafish and mussels; zebrafish exposed to BPA for 96 h at environmentally relevant concentrations (220, 1180, and 1500 ng/L) showed an increased production of reactive oxygen species (ROS) and a significant reduction in AChE activity [11]. Chronic exposure to BPA (0.25, 1, 2, and 5 μg/L, 28d) caused concentration-dependent inhibition of AChE in the date mussel, *Lithophaga lithophaga* [37]. One can envisage that in *Ciona*, as in the other species examined so far, AChE inhibition may cause the persistence of neurotransmitters in the neuronal cleft, which in turn can induce an altered pathway of branchial sac contraction in response to different stimuli, thus influencing an organism’s behavior. From this perspective, further studies are fundamental to link the disruption/inhibition of ChE vs. ASCh activity caused by BPA to predict behavioral endpoints. We found that BPA inhibits ChE and ASCh activity in the V, T, and S, while PS NPs had no effect on ChE enzyme activity alone or in combination with BPA. This is consistent with previous studies on *Ciona* larvae, in which no effect of amino-modified PS NPs (PS-NH_2_) was observed upon in vivo exposure up to µg/mL (22 h post-fertilization) [27]. Co-exposure to PS NPs and BPA resulted in a 50% inhibition of ChE activity compared to ASCh, consistent with the effect of BPA alone (Table 2). DLS analysis of the ζ-potential showed no significant changes between PS NPs alone in mQW and combined with BPA in the ChE reaction buffer after 15 min of incubation (Table 1), thus indicating no interaction between PS NPs and BPA within this timeframe. The short incubation time (15 min) may not have allowed sufficient interactions between BPA and the PS NPs to induce notable changes in surface charge. Studies have shown that interaction time plays a key role in surface modifications, and longer exposure periods could potentially lead to different outcomes [38]. Additionally, the buffer’s composition itself can stabilize the NPs by creating an ionic environment that shields surface interactions, explaining the lack of variation in the ζ-potential. The presence of ions in the buffer can also enhance colloidal stability, reducing electrostatic interactions between BPA and PS NPs. This was similarly observed in studies examining NPs’ interactions in complex media, where surface charge often remains stable in short-term exposures despite the presence of other compounds [38,39]. Therefore, it is plausible that under different timeframes or conditions, BPA could influence PS NPs more significantly, warranting further investigation. On the other hand, in vivo exposure studies, such as the one by Chen et al. [3], showed that combined exposure to PS NPs and BPA led to a significant uptake of BPA in the head and viscera of zebrafish, along with significant inhibition of AChE activity. Therefore, waterborne exposure lasting for 3 days is enough for BPA and PS NPs’ ability to interact, leading to synergistic effects. However, such interaction could be strongly abolished by the presence of seawater, as shown in our recent study in which the effects on pigmentation in *Ciona* larvae were visually the same upon exposure to BPA alone or in combination with PS NPs. This suggested that no interaction was taking place between BPA and PS NPs in the seawater media, unlike in freshwater media where a carrier role of PS NPs towards BPA has been hypothesized [3]. The absence of an interactive effect of PS NPs towards BPA in seawater, compared to freshwater, was attributed to the high ionic strength of seawater, which can trigger the sorption surface properties of PS NPs as confirmed by the significant changes in the NPs’ surface charges shown by ζ-potential values. Thus, a further important variable factor concerns the media used for the experiments [21,38].

## 5. Conclusions

To the best of our knowledge, our study provides the first evidence of ChE vs. ASCh activities in the tissues (V, T, and S) of adults of *Ciona*. Sensitivity towards BPA was confirmed as an inhibitor of ChE vs. ASCh activities, while no effects have been observed for PS NPs alone or combined with BPA. Therefore, when combined as in a realistic exposure scenario, BPA neurotoxicity is occurring regardless of the presence of PS NP. The concentration-dependent inhibition of ChE vs. ASCh activity by BPA underscores its potential neurotoxic effects on ascidians at the adult stage, as already observed at the embryonic stage with depigmentation and abnormal development [21].

Overall, this study contributes to the understanding of BPA neurotoxicity in ascidian by targeting ChE vs. ASCh activities in several organs and no further effects from the synergistic exposure to nanoplastics is expected. Indeed, nanoplastic–molecule interactions must be considered as case-by-case studies, as well as the need to perform in vivo studies for setting more realistic scenarios in which interactions could occur at levels other than the AChE binding site.

## Figures and Tables

**Table 1:** ζ-potential measured by Dynamic Light Scattering (DLS) of non-functionalized PS NPs (50 µg/mL) alone in mQW and combined with BPA (0.69 mM) in ChE reaction buffer, at time 0 (T_0_) and after 15 min of incubation (T_15_). Values are shown as mean ± standard deviation.

	ζ-Potential (mV)
mQW	−51 ± 5
ChE reaction buffer	−49.1 ± 1.78
ChE reaction buffer + BPA (T_0_)	−50.8 ± 1.72
ChE reaction buffer + BPA (T_15_)	−47.3 ± 1.57

**Table 2:** Optimization of ChE vs. ASCh in viscera (V) and sensitivity towards the selected AChE inhibitor BW. ChE activities are expressed as nmol/min/mg proteins and shown as mean ± standard deviation.

Viscera (V)			
**Extract (µL)**	**Substrate ASCh (mM**)	**ChE**	
40	0.24	58.2 ± 21.6	
60		76.8 ± 7.8	
80		81.8 ± 3.2	
100		126 ± 8.7	
**Extract (µL)**	**Substrate ASCh (mM)**	**ChE**	
100	0.24	90.3 ± 16.2	
	0.36	136.6 ± 26.2	
	0.48	201.7 ± 14.8	
**Extract (µL)**	**Substrate ASCh (mM)**	**ChE**	**BW284C51** **(1 µM) residual activity**
100	0.48	95.1 ± 8.6	10.8 ± 2.4

**Table 3:** Optimization of ASCh vs. ChE in siphons (S) and tunic (T) of Ciona. ChE vs. ASCh activities are expressed as nmol/min/mg proteins and shown as mean ± standard deviation.

Siphons (S)			Tunic (T)
**Extract (µL)**	**Substrate ASCh (mM)**	**ChE**	**ChE**
40	0.24	58.2 ± 21.6	1.04 ± 0.7
60		76.8 ± 7.8	0.18 ± 0.6
80		81.8 ± 3.2	11.6 ± 2.2
100		126 ± 8.7	0.18 ± 0.6
**Extract (µL)**	**Substrate ASCh (mM)**	**ChE**	**ChE**
100	0.48	9.13	3.78

**Table 4:** Percentage of ChE vs. ASCh activities inhibition vs. controls (Ctrl).

**BPA Alone (mM)**	**%ChE Inhibition**
Ctrl	0
0.01	26.8
0.2	48.42
0.69	50.86
**PS NPs Alone (mM)**	**%ChE Inhibition**
Ctrl	0
0.0096	0
0.096	0
**BPA + PS NPs (mM)**	**%ChE Inhibition**
Ctrl	0
0.69 + 0.096	46.34

## Data Availability

The data presented in this study are available in this article.

## Appendix A

**Supplementary informations—Method used to calculate the number of PS NP per mL**

Nanoparticle diameter (from TEM image) = 33 nm
Radius = 16.5 nm
Polystyrene density = 1.05 g/cm^3^
The surface area of a single particle: 4πr ^2^ → 4 × π × 16.5^2^ = 3419.46 nm^2^
The volume of a single particle: 4/3πr^3^ → 4/3 × π × 16.5^3^ = 18,816.56 nm^3^
Weight of a single particle (convert 18,816.56 nm^3^ to cm^3^):
Density*number of particles → 1.05 × 1.88 × 10^−17^ = 1.974 × 10^−17^ g
Number of particles/mL (convert particle concentration to g/mL and divide by the weight of a single particle):
1 µg/mL → 1 × 10^−6^ g/mL → (1 × 10^−6^)/(1.974 × 10^−17^) = 0.506 × 10^11^ particles/mL
10 µg/mL → 1 × 10^−5^ g/mL → (1 × 10^−5^)/(1.974 × 10^−17^) = 0.506 × 10^12^ particles/mL
Calculation of number of particles in 160 µL used for the in vitro study:
1 µg/mL → 8.096 × 10^9^ particles
10 µg/mL → 8.096 × 10^10^ particles
